# Accurate multi-behavior sequence-aware recommendation via graph convolution networks

**DOI:** 10.1371/journal.pone.0314282

**Published:** 2025-01-07

**Authors:** Doyeon Kim, Saurav Tanwar, U. Kang

**Affiliations:** Seoul National University, Seoul, Republic of Korea; Augusta University, TAIWAN

## Abstract

*How can we recommend items to users utilizing multiple types of user behavior data?* Multi-behavior recommender systems leverage various types of user behavior data to enhance recommendation performance for the target behavior. These systems aim to provide personalized recommendations, thereby improving user experience, engagement, and satisfaction across different applications such as e-commerce platforms, streaming services, news websites, and content platforms. While previous approaches in multi-behavior recommendation have focused on incorporating behavioral order and dependencies into embedding learning, they often overlook the nuanced importance of individual behaviors in shaping user preferences during model training. We propose MBA (Multi-Behavior sequence-Aware recommendation via graph convolution networks), an accurate framework for multi-behavior recommendations. MBA adopts a novel approach by learning embeddings that capture both the dependencies between behaviors and their relative importance in influencing user preferences. Additionally, MBA employs sophisticated sampling strategies that consider the sequential nature of behaviors during model training, ensuring that the model effectively learns from the entire behavioral sequence. Through extensive experiments on real-world datasets, we demonstrate the superior performance of MBA compared to existing methods. MBA outperforms the best competitor, achieving improvements of up to 11.2% and 11.4% in terms of HR@10 and nDCG@10, respectively. These findings underscore the effectiveness of MBA in providing accurate and personalized recommendations tailored to individual user preferences.

## Introduction

*How can we recommend items to users utilizing multiple types of user behavior data?* Recommender systems [1–4] are becoming increasingly important across various applications for providing personalized suggestions to users. They play a pivotal role in organizing and customizing data, helping users discover relevant content, products, or services amidst the overwhelming volume of information [5, 6]. However, capturing user preferences in recommender systems is challenging due to the limited interactions users have with items related to the target behavior, such as *buy*. To address this challenge, recent research has introduced multi-behavior recommendations [7–10], aiming to effectively capture user-item preferences by leveraging various behaviors exhibited by users, including *view*, *cart*, and *buy*.

In e-commerce platforms, user behaviors often unfold in structured sequences. For instance, users typically start by browsing (*view*), then may proceed to add items to their cart (*cart*), and finally make a purchase (*buy*). These sequences are not random but rather follow a logical flow, with each behavior playing a specific role in the overall process. Consider the sequence *view→cart→buy*. Here, each behavior contributes differently to the eventual purchase decision. While *view* indicates initial interest, *cart* signifies a higher level of intent, as users actively select items for potential purchase. Finally, *buy* represents the culmination of the user’s decision-making process, indicating a confirmed purchase. The influence of each behavior on the target behavior (*buy*) varies. For instance, *cart* has a more direct influence on *buy* compared to *view*. This implies that users who have added items to their cart are more likely to proceed with a purchase compared to those who have merely viewed items. Understanding these behavioral sequences is crucial for accurate recommendation systems. A recommendation system that incorporates behavioral sequences better reflects user preferences. For example, items that are frequently added to the cart after being viewed should be prioritized in recommendations, as they are more likely to lead to *buy*.

E-commerce users have varying behaviors for choosing the next action of a given action. Table 1 shows the transfer probability between different behaviors within three real-world e-commerce platforms (see Experiments section for details). The probability *P*(*B*′∣*B*) represents the likelihood of a subsequent behavior *B*′ given a prior behavior *B*. In Tmall dataset, for instance, the low probability of transitioning from *view* to *cart* (*P*(*cart*∣*view*) *= 0.1%*) indicates that few users proceed to add items to their cart after viewing them. This suggests that the transition from browsing to cart addition is relatively rare, potentially due to factors such as browsing for comparison or lack of immediate purchase intent. In Jdata dataset, *P*(*buy*∣*view*) = 12.0% while *P*(*buy*∣*cart*) = 50.8%. This means that there is a greater tendency for items in *cart* to be bought than items in *view*, which shows that the behavior close to *buy* is more related to user preferences. These findings underscore the significance of understanding behavior sequences and their implications for user preferences. While previous research [9, 11] has explored the order of behaviors in recommendation models, many fail to account for the varying importance of each behavior within different sequences.

**Table 1:** The ratio of the behavior transfer between two behaviors.

Dataset	*P*(*cart*∣*view*)	*P*(*buy*∣*view*)	*P*(*buy*∣*cart*)
**Tmall** ^1^	0.1%	8.2%	10.9%
**Jdata** ^2^	2.4%	12.0%	50.8%
**Beibei** ^3^	9.2%	9.3%	76.9%

^1^ https://tianchi.aliyun.com/dataset/649

^2^ https://global.jd.com

^3^ https://www.beibei.com

In this paper, we propose MBA (Multi-Behavior sequence-Aware recommendation via graph convolution networks), a novel approach designed to address the challenges of multi-behavior recommendation tasks. MBA leverages graph convolution networks (GCNs) to effectively capture the intricate dependencies between user behaviors within recommendation systems. One key aspect of MBA is its ability to learn embeddings that encode the order and importance of behaviors within sequences. Considering the sequential nature of user interactions, MBA better understands the progression from initial behaviors like *view* to final behaviors like *buy*, capturing nuanced patterns that reflect user preferences. Moreover, MBA tackles the issue of data sparsity by utilizing interaction information from previous behaviors. This enables the model to leverage historical user actions, even when direct interactions with target behaviors are limited, thereby improving recommendation accuracy. Additionally, MBA incorporates a mechanism for learning the relative importance of different behaviors in contributing to the prediction of target behaviors. Furthermore, MBA is capable of accommodating diverse user preferences within behavior sequences during model training. Considering the varying significance of behaviors in different contexts, MBA tailors its recommendations to better align with individual user interests and preferences.

Our main contributions are summarized as follows:

- **Method.** We propose MBA, an accurate method for multi-behavior recommendation. MBA incorporates several innovative components, including the exploitation of interaction transfer between behaviors, a behavior-aware attention network, and a novel sampling technique for Bayesian Personalized Ranking (BPR).
- **Performance.** Through extensive experiments on three benchmark datasets, we demonstrate the superior performance of our proposed method. Compared to existing state-of-the-art approaches, MBA achieves notable improvements, with up to 11.2% and 11.4% enhancement in HR@10 and nDCG@10 metrics, respectively. These results underscore the effectiveness of MBA in delivering more relevant and personalized recommendations to users across various scenarios.
- **Analysis.** In addition to evaluating the performance of MBA, we conduct thorough analyses to investigate the impact of leveraging multi-behaviors in different configurations. By exploring various combinations and orders of behaviors, we gain valuable insights on how different factors influence recommendation outcomes.

The rest of the paper, we first explain the preliminaries about multi-behavior recommender systems and review existing multi-behavior recommender systems. Then we describe the proposed method MBA in detail and present experimental results to evaluate MBA. We summarized the symbols frequently used in this paper in Table 2. The code and datasets are available at https://github.com/snudatalab/MBA.

**Table 2:** Description of symbols.

Symbol	Description
U	A set of users
I	A set of items
B	A set of behaviors
*K*	Number of behaviors in *B*
**Y**	A set of user-item interaction matrices
**Y**_*k*_	User-item interaction matrix of *k*-th behavior
**A**_*k*_	Adjacency matrix of *k*-th behavior
**E** ^(*k*, *l*)^	Embedding matrix of *l*-th layer under *k*-th behavior

## Related works

### Multi-behavior recommendation

Multi-behavior recommendation uses various types of user-item interactions for the recommendation. It has gained increasing attention due to its effectiveness in addressing data sparsity and enhancing recommendation performance. To improve the prediction ability of target behavior, many works focus on capturing signals from other types of behaviors. Early approaches to multi-behavior recommendation were based on traditional techniques, such as extending matrix factorization (MF) to multiple matrices [8, 12, 13]. For example, Ajit et al. [12] proposed a Collective Matrix Factorization (CMF) which decomposes multiple matrices simultaneously by sharing parameters among factors. Zhao et al. [13] extended CMF to perform matrix factorization of multiple behaviors by sharing user or item embeddings. DaConA [8] incorporates a data context adaptation layer by sharing latent vectors and learns non-linear relations between them via a neural network. In addition, some works have designed new sampling strategies to exploit different behaviors [14, 15]. Loni et al. [14] extended Bayesian Personalized Ranking (BPR) [7] by designing a negative sampling strategy to sample user-item interaction data with different behaviors. Guo et al. [15] utilized the similarity of items to generate samples from multiple auxiliary behaviors. The major limitation of these methods is the lack of exploration of the relationship among behaviors.

In this work, we model different behaviors in the form of cascading GCN blocks by effectively exploring the preference relation between behaviors. We propose to refine the user embedding based on cascading GCN blocks, corresponding to the decision-making process of users.

### Graph Convolution Network for recommendation

Graph Convolution Network (GCN) models have succeeded in a variety of applications [11, 16–20]. The basic concept behind GCN involves continuously updating the representation of a specific node by aggregating data from its neighbors within the graph. Due to its strong capability of representation, GCN has also been widely applied in recommender systems [17, 20, 21], since relations between users and items are naturally represented by graphical structures. Wang et al. [17] exploit the user-item graph structure by propagating embeddings which leads to the expressive modeling of high-order connectivity of the graph. He et al. [20] proposed LightGCN which removes feature transformation and nonlinear activation from GCN but neighborhood aggregation, and improved recommendation performance.

Moreover, GCN-based models are also widely adopted in multi-behavior recommendation tasks [11, 18, 19, 22]. For example, Chen et al. [18] formulate the multi-behavior recommendation in a heterogeneous graph with nodes of users, items, and edges of behaviors. Graph Heterogeneous Multi-Relational Recommendation (GHCF) [19] leverages GCN to model high-hop heterogeneous user-item interactions and improve the representation of users and items along with their relationships. Multi-Behavior Graph Convolutional Network (MBGCN) [11] is constructed with a unified graph to represent multi-behavior data and learn the influence strength to the target behavior. Graph Neural Multi-Behavior Enhanced Recommendation (GNMR) [22] explores multi-behavior dependencies through recursive embedding propagation on a unified graph. It employs a relation aggregation network to accurately represent the diverse interactions within the graph. Most existing models focus on leveraging GCNs to capture relationships between users and items in multi-behavior scenarios. However, there is a lack of in-depth investigation into the order of behaviors. Behavioral order plays a crucial role in determining the influence of one behavior on another, which is the main concern of our MBA model.

In our work, we propose cascading GCN blocks, which not only effectively extract the preference signal of each type of behavior but also refine user embedding by extracting useful information from the signal learned in each behavior.

### Behavior sequence-aware recommendation

Behavior sequence-aware recommendation [9, 10, 23] aims to explore the dependencies between multiple behaviors for embedding learning. This approach involves taking common behavior sequences and learning the embeddings of each behavior continuously to capture behavioral dependencies. Cascading Residual Graph Convolutional Network (CRGCN) [9] utilizes a cascading GCN structure to refine user preferences continuously and retain prior behavioral features as initial embeddings for the following behavior. On the other hand, Multi-Behavior Recommendation Model with Cascading Graph Convolution Networks (MB-CGCN) [10] transfers behavior features learned by LightGCN from the previous behavior to the next behavior after a feature transformation step and aggregates all behavior embeddings for the final prediction. Efficient Noise-Decoupling for Multi-Behavior Sequential Recommendation (END4Rec) [23] addresses different noise types of long user behavior sequences and captures intricate behavior patterns. However, existing models fail to consider the importance of different behaviors and treat all behaviors equally. As a result, the influence of specific behaviors on predicting the target behavior is not be adequately captured. Also, they still have data sparsity problems as interactions become sparse in the order of *view* > *cart* > *buy*. Data sparsity, especially in scenarios where many user-item pairs are unobserved, significantly impact the model’s performance if not properly accounted for.

In our work, we address the sparsity issue of target behaviors by sharing user-item interaction information between behaviors. We consider the relative importance of behaviors since each behavior contributes differently to the target behavior.

## Proposed method

We propose MBA (Multi-Behavior sequence-Aware recommendation via graph convolution networks), an accurate method for multi-behavior recommendation. The main challenges and ideas of MBA are as follows:

1. **How can we solve the problem that interaction decreases depending on the order of behaviors?** We share interaction information between behaviors. We build cascading behavior blocks that transfer node embeddings with edges to the next behaviors.
2. **How can we exploit the difference of importance in behaviors for the target behavior prediction?** We aggregate embeddings learned from all behaviors with attention weights.
3. **How can we train the model considering the behavior sequence?** We propose a novel BPR loss function that compares user preferences within behavior sequences by sampling items from different behaviors.

**Algorithm 1:** MBA: Multi-Behavior Sequence-Aware Recommendation

**Input:** The user-item interaction data of *K* types of behaviors {**Y**_1_, **Y**_2_, …, **Y**_*K*_}, for a user set U and an item set I.

**Output:** The probability that a user *u* will interact with an item *i* under the *K*-th behavior, i.e., target behavior.

1: **Cascading GCN blocks.** Learn the user and item embeddings using LightGCN for each behaviors in a defined order. More specifically, the embeddings learned from a previous behavior will be delivered to facilitate the next behavior’s embedding learning.

2: **Embedding aggregation.** Aggregate the embeddings learned from each behavior with attention weights for the target behavior prediction.

3: **Sampling items.** Randomly sample user *u*’s interacted item *p*. If the behavior with the last interaction of item *p* is *B*_*p*_, sample item *n* with the last interaction in the behavior before *B*_*p*_.

4: **Training the model.** Train the model by maximizing the the embedding difference between item *p* and item *n*. Predict a probability that a user *u* will take a target behavior (e.g., *buy*) to an item *i*. Generate the recommendation list by sorting items based on the probability in descending order.

**Fig 1:**
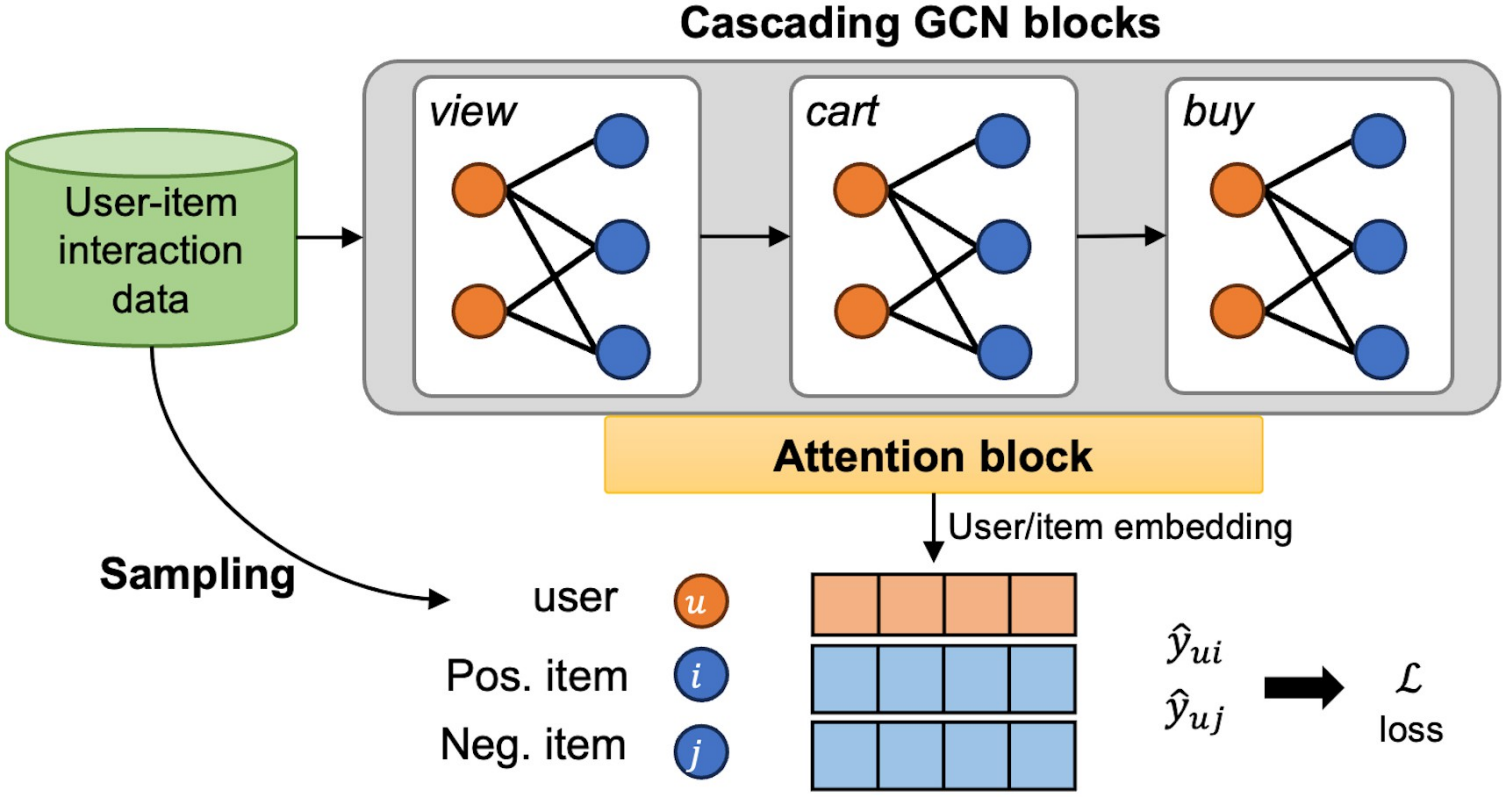
Overview of MBA.

Algorithm 1 shows the overall process of MBA. Given the various interaction data for users and items, cascading GCN blocks represent their embeddings for each behavior (line 1 in Algorithm 1). The fundamental idea is 1) to leverage cascading LightGCN to extract features from various behaviors and 2) to utilize dependencies within the behavior chain to aid in learning features for subsequent behaviors. The output embeddings for each GCN block then go through the attention block to learn the importance of the behavior to target behavior prediction (line 2 in Algorithm 1). We sample items from different behaviors where relatively positive and negative items exist (line 3 in Algorithm 1), and train the model by considering the ranking of the items (line 4 in Algorithm 1). Fig 1 shows the illustration of MBA.

### Cascading GCN blocks

The goal of the cascading GCN blocks is to extract user preferences from individual behaviors and capture the cascading relations of user preferences among behaviors to comprehensively understand user preferences. The primary concept involves starting with basic features, such as initialized user and item embeddings, and iteratively refining them by incorporating behavioral features learned from each behavior type.

Cascading GCN blocks mainly consists of a chain of LightGCN modules, with each LightGCN module dedicated to learning user and item embeddings for a specific behavior. Within this chain, the embedding obtained from a previous LightGCN module is used as input features for users and items in the subsequent LightGCN module. Moreover, interaction information between connected behaviors is combined to enhance the information available for latter behaviors.

#### Interaction sharing between behaviors

As mentioned earlier, different user behaviors often indicate various aspects of preferences towards an item. Moreover, the behaviors interacting with items in a certain order reveal user preferences at different degrees. Our goal is to continuosly enhance user preferences by incorporating all behavioral features and leveraging the connections between different behaviors. In a sequence of behaviors, later behaviors often convey stronger signals or more accurate indications of user preference compared to earlier ones. Consequently, embeddings learned from previous behaviors are good initializations for the next behavior’s embedding learning, which forms the core concept of our cascading GCN structure. For example, the embeddings learned from the first behavior (i.e., *view*) are directly used as the initialized embeddings in the next behavior (i.e., *cart*) for embedding learning; and the same for the embedding learning of the last behavior (i.e., *buy*). Overall, this approach facilitates the gradual improvement of user preference modeling by leveraging the strengths of different behaviors and effectively utilizing available data.

By leveraging embeddings learned from the previous behavior, we enhance the information available for subsequent behaviors by transferring interaction details between connected behaviors. Each behavior’s adjacency matrix captures the interactions between users and items relevant to that behavior. To share interaction data between two behaviors, we use the summation for the matrices. Summing these matrices allows for comprehensive consideration of interactions among all behaviors in the entire network. Consequently, the model leverages not only the information from each behavior but also the relationships among different behaviors for predictions. Also, it helps tackle the issue of decreasing interaction strength depending on the sequence of behaviors (e.g., *view>cart>buy*). Sparse interactions lead to inadequate learning of user and item representations since these embeddings are derived from interactions.

Given the adjacency matrices **A**_1_, …, **A**_*K*_ for all behaviors, where $A_{k}=[\begin{array}{cc} 0 & Y_{k}\\ {\left(Y_{k}\right)}^{⊤} & 0 \end{array}]\in R^{\left(|U|+|I|)\times (|U|+|I|\right)}$ and user-item interaction matrix $Y_{k}\in R^{\left|U|\times |I\right|}$ for *k*-th behavior, we define a new matrix ${\hat{A}}_{k}\in R^{\left(|U|+|I|)\times (|U|+|I|\right)}$ as follows:

$${\hat{A}}_{k}=\{\begin{array}{ll} A_{1} & k=1\\ A_{k}+A_{k-1} & \text{otherwise} \end{array}$$ (1)

The first behavior solely utilizes its own adjacency matrix since it doesn’t have a previous behavior, while other behaviors combine their own adjacency matrix with that of the previous behavior. The transmission of information between behaviors yields two main advantages: firstly, from a user preference modeling perpective, it facilitates continuous refinement of embeddings, leading to more precise depiction of user preferences. Secondly, in addressing data sparsity concerns, it enables better utilization of data that has not yet transitioned into target behaviors, thereby aiding in learning user preferences and alleviating challenges associated with cold-start users to some extent.

#### Single-behavior modeling

In order to learn representations of users and items for each behavior, we adopt LightGCN [20] which recursively integrates the embedding information from neighboring nodes. In LightGCN, user and item entities are represented as nodes within the graph structure. Each node’s representation is updated by aggregating information from its neighboring nodes in the graph. This aggregation process is repeated recursively over multiple layers, allowing nodes to gather and incorporate information from distant parts of the graph. Given the embedding matrix $E^{\left(k,l\right)}\in R^{(|U|+|I|)\times d}$ for *l*-th layer under *k*-th behavior with embedding size *d*, the node representation obtained from a single GCN layer is defined as:

$$\begin{array}{c} E^{\left(k,l+1\right)}=B_{k}E^{\left(k,l\right)} \end{array}$$ (2)

where $B_{k}=D_{k}^{-\frac{1}{2}}{\hat{A}}_{k}D_{k}^{-\frac{1}{2}}$ is the normalized adjacency matrix and **D**_*k*_ is the diagonal degree matrix of ${\hat{A}}_{k}$. After *L* layers of propagation, we obtain *L* + 1 layers of embeddings for each behavior. We average these embeddings to obtain the final embeddings of each behavior as follows:

$$\begin{array}{c} E^{k}=\sum_{l=0}^{L}\frac{1}{L+1}E^{\left(k,l\right)} \end{array}$$ (3)

The final embeddings for user *u* and item *i* of *k*-th behavior are represented as $e_{u}^{k}$ and $e_{i}^{k}$, which are the row vectors corresponding to user *u* and item *i* in **E**^*k*^, respectively.

### Behavior-aware attention network

To exploit the varying importance of behaviors in target behavior prediction, we leverage the observation that different behaviors possess different levels of informativeness for learning user representations. For example, *buy* behavior is more informative than *view* or *cart* behavior. While a *view* action indicates a user’s initial interest in an item, a *cart* action suggests a higher level of engagement, indicating a potential intention to purchase. However, it’s often the *buy* behavior that offers the most valuable information, as it directly reflects a user’s decision to make a purchase.

Thus, identifying the important behaviors of users has the potential to yield more informative user representations. For instance, we assign higher weights or attention to behaviors like *buy* when learning user representations, as they are more indicative of user preferences and intentions. Conversely, behaviors like *view* or *cart* receive lower weights, reflecting their lesser impact on understanding user preferences. To achieve this, we propose employing a behavior-aware attention network to select important behaviors within the global context of behavioral order. An attention network refers to a neural network architecture that includes an attention mechanism [24]. The attention mechanism allows the network to selectively focus on certain parts of the input data, enhancing its ability to process and understand complex patterns [25]. This is typically achieved through a set of learnable parameters that compute attention scores for each element of the input. In MBA, the attention weight *α*^*k*^ for the user *u*’s vector for the *k*-th behavior is given as follows:

$$\begin{array}{c} \alpha^{k}=\frac{\text{exp}(a^{k})}{\sum_{n=1}^{K}\text{exp}\left(a^{n}\right)} \end{array}$$ (4)

where $a^{k}=q^{⊤}\cdot \text{tanh}\left(W\times e_{u}^{k}+v\right)$. Here, $W\in R^{d\times d}$ and **v** are parameters, **q** represents the attention query vector, and *K* denotes the number of behaviors.

The final user representation is obtained by summing the user representations of all behaviors weighted by their attention weights, i.e., $u=\sum_{k=1}^{K}\alpha^{k}e_{u}^{k}$. On the other hand, the fusion of multiple behaviors for an item differs from that of a user since the features of items are static. Therefore, we simply sum all embeddings of item *i* from different behaviors to obtain the final representation of item, i.e., $r_{i}=\sum_{k=1}^{K}e_{i}^{k}$.

Finally, the model prediction is defined as the inner product of the user and item representations, i.e., ${\hat{y}}_{ui}=u^{T}\cdot r_{i},$ which serves as a prediction score for the target behavior recommendation.

### Model training

We adopt Bayesian Personalized Ranking (BPR) loss [7] for training our model, which is widely used in the recommendation system [2, 10, 13]. It emphasizes the relative order between observed and unobserved user-item interactions. It asserts that observed interaction, which is informative for user’s preference learning, should have a higher prediction score than unobserved ones.

**Fig 2:**
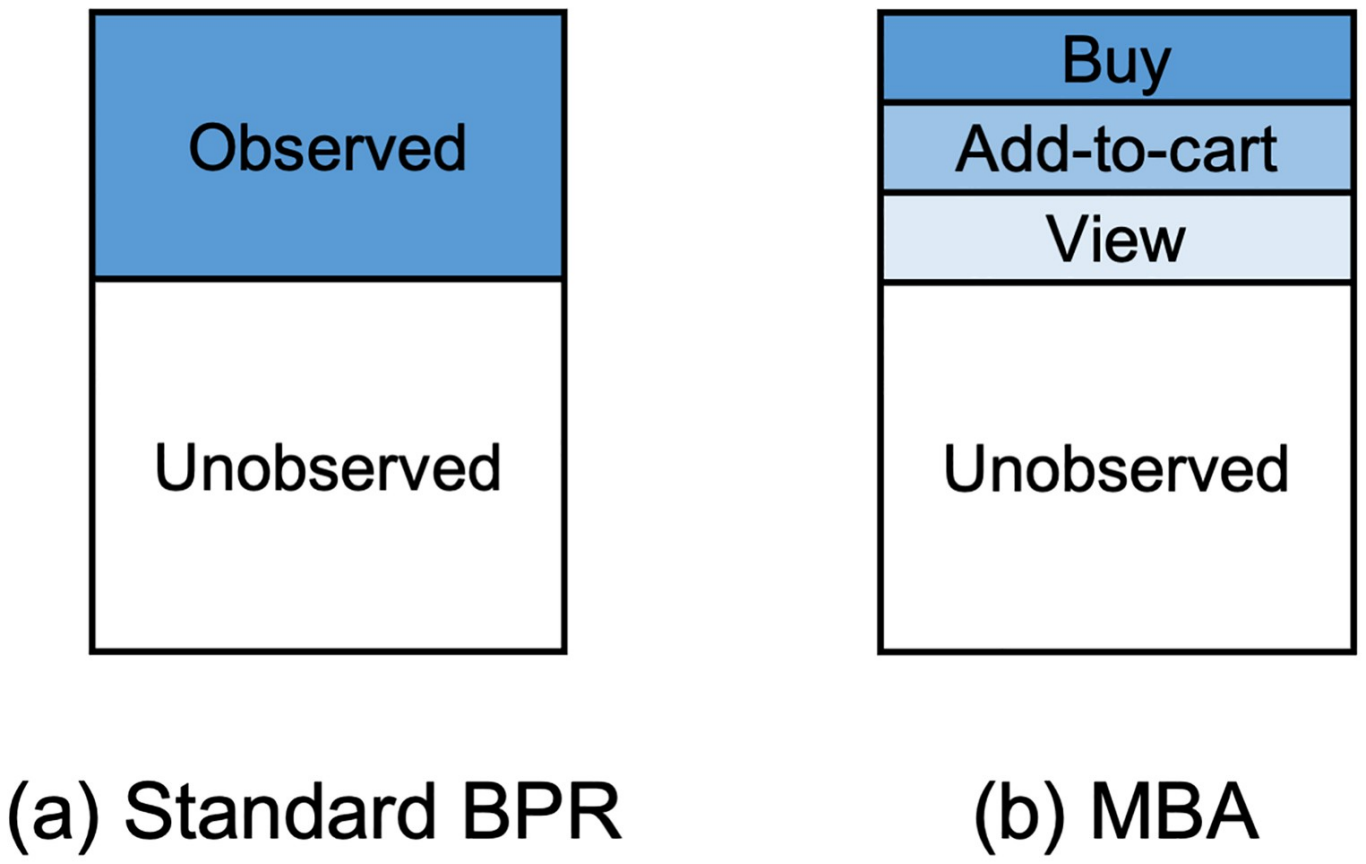
Sampling user-item pairs in the standard BPR (a) and MBA (b). Sampling user-item pairs in the standard BPR (a) and MBA (b). Behaviors are stacked from bottom to top in behavior order. The higher the behavior is positioned, the stronger the preference exhibited by the pairs within.

The BPR loss is minimized during model training, with the objective of optimizing the model’s performance. Specifically, for a given user set U and item set I, the BPR loss is defined as:

$$\begin{array}{c} L=\sum_{(u,i,j)\in O}-\text{log}\sigma \left({\hat{y}}_{ui}-{\hat{y}}_{uj}\right) \end{array}$$ (5)

for $O=\{\left(u,i,j\right)|u\in U,i\in I_{u},j\notin I_{u}\}$, where *i* is a positive item, *j* is a negative item. *I*_*u*_ is the set of items that user *u* has interacted with. The standard BPR considers observed user-item pairs solely in the target behavior (e.g., *buy*) as illustrated in Fig 2(a). However, it overlooks observed user-item pairs in other behaviors (e.g., *view* and *cart*). Notably, there exists a preference difference between items in *view, cart, buy* and the unobserved category. Typically, users tend to add items to their carts before making a purchase, suggesting that the distance between a bought item and a carted item is closer than that between a bought item and an unobserved one.

As shown in Fig 2(b), MBA assigns different levels to different behaviors, thereby indicating the significance of each type of behavior during the training phase.

#### Behavior-based sampling

To explore different behavior sequences in model training, we compare user preferences for sampled items across different behaviors. We sample positive items from behaviors with high user preferences, and negative items from less informative behaviors. Therefore, we enhance BPR by sampling positive and negative items according to the behavior sequences to explore user preferences in behaviors. Let $B=\{B_{1},...,B_{K}\}$ represent a given ordered set of *K* behaviors, and the unobserved interaction is also considered as a behavior *B*_0_. We make a positive item have a latter behavior than a negative item since items with the latter behavior reflect stronger user preferences. We define *B*_*p*_ as the behavior of positive item *i* and *B*_*n*_ as the behavior of negative item *j*. Then, *B*_*n*_ comes before *B*_*p*_ in the behavior sequence, i.e., *n* < *p*. When a user’s interacted item appears in all of *view*, *cart*, and *buy*, it is recognized as an item within *buy*. Also, if *B*_*p*_ = *buy*, *B*_*n*_ is one of *view*, *cart* or the unobserved interaction *B*_0_.

While sampling positive items, we aim to sample items with stronger user preferences. We assign a higher preference to a behavior if it appears later in a behavior sequence, and a lower preference otherwise. Given all the observed user-item pairs *S*, we sample positive items using sampling distribution *P*(*u*, *i*, *B*_*p*_) = *P*(*u*, *i*|*B*_*p*_)*P*(*B*_*p*_), where *P*(*u*, *i*|*B*_*p*_) is a uniform distribution over user-item pairs in behavior *B*_*p*_ and *P*(*B*_*p*_) is the sampling distribution of behavior *B*_*p*_. We define probability *P*(*B*_*p*_) as:

$$\begin{array}{c} P\left(B_{p}\right)=\frac{w_{B_{p}}\left|S_{B_{p}}\right|}{\sum_{Q\in B}w_{Q}\left|S_{Q}\right|} \end{array}$$ (6)

where $w_{B_{p}}$ is the weight of behavior *B*_*p*_, and $S_{B_{p}}$ contains user-item pairs in behavior *B*_*p*_. *P*(*B*_*p*_) is designed to sample a behavior following the data distribution, while adjusting the weights *w*_*i*_ so that a latter behavior has a higher preference. We use *w*_1_ (*view*) = 1, *w*_2_ (*cart*) = 2, and *w*_3_ (*buy*) = 3 which show a good performance.

Given positive user-item pair (*u*, *i*) and their behavior *B*_*p*_, we sample a negative item *j* with behavior *B*_*n*_. We sample *B*_*n*_ from unobserved interaction *B*_0_ or behaviors which are prior to *B*_*p*_ in the behavior sequence, denoted as *B*_*n*_ ≺ *B*_*p*_, making *B*_*n*_ relatively negative than *B*_*p*_. We use *P*(*j*, *B*_*n*_|*u*, *B*_*p*_) as the uniform distribution of negative item *j* and its corresponding behavior *B*_*n*_. Similar to the positive sampling, the distribution of negative sampling is *P*(*j*, *B*_*n*_|*u*, *B*_*p*_) = *P*(*j*|*u*, *B*_*p*_, *B*_*n*_)*P*(*B*_*n*_|*u*, *B*_*p*_), where *P*(*j*|*u*, *B*_*p*_, *B*_*n*_) is the uniform distribution over user-item pairs $S_{B_{n}}$, and *P*(*B*_*n*_|*u*, *B*_*p*_) is the sampling distribution of behavior *B*_*n*_. We define *B*_*n*_ as:

$$\begin{array}{c} P\left(B_{n}|u,B_{p}\right)=\frac{|S_{B_{n}}|}{\sum_{Q≺B_{p}}\left|S_{Q}\right|} \end{array}$$ (7)

since we want to give a higher sampling probability to a behavior *B*_*n*_ the earlier it appears in a behavior sequence, and an early behavior (e.g., view) has a higher frequency than a latter behavior (e.g., buy) in general.

## Experiments

We perform experiments to answer the following questions:

Q1. **Performance.** How accurately does MBA predict the target behavior compared to the competitors?
Q2. **Ablation study.** How does each module in MBA affect the recommendation performance?
Q3. **Analysis.** How do the number of behaviors and behavioral order affect MBA?

### Experimental settings

We build all models using the Pytorch framework. All the models are trained and tested on a machine with GeForce GTX 1080 Ti GPU.

#### Dataset

To evaluate the performance of our model, we conduct experiments on three real-world datasets generated from user logs in different e-commerce platforms in China.

- **Tmall.** This dataset is collected from Tmall, one of the largest e-commerce platforms in China. It contains 41,738 users and 11,953 items with 4 types of behaviors, *i*.*e*., *view*, *collect*, *cart*, and *buy*. On the Tmall platform, users can buy the item directly after viewing, or add it to the cart before purchasing, or they may just click on the collection instead of the *buy* behavior.
- **Jdata.** This dataset is collected from JD, a comprehensive online retailer in China. This dataset contains 93,334 users and 24,624 items with 4 types of behaviors, *i*.*e*., *view*, *collect*, *cart*, and *buy* within the period from 2018/02/01 to 2018/04/15. The behavior is similar to that of Tmall.
- **Beibei.** This dataset is collected from Beibei, an e-commerce platform in China. This dataset contains 21,716 users and 7,977 items with three types of behaviors, including *view*, *cart*, and *buy* within the period from 2017/06/01 to 2017/06/30.

For the three datasets, we follow the previous work to merge the duplicated user-item interactions by keeping the earliest one [9, 11]. The statistical information of the three datasets used in our experiments is summarized in Table 3.

**Table 3:** Summary of multi-behavior datasets. ***view, collect, cart,*** and ***buy*** are the behavior types in interactions.

Dataset	Users	Items	Interactions	*view*	*collect*	*cart*	*buy*
**Tmall** ^1^	41,738	11,953	2,218,436	1,813,498	221,514	1,996	181,428
**Jdata** ^2^	93,334	24,624	1,916,589	1,600,973	39,663	41,262	234,691
**Beibei** ^3^	21,716	7,997	3,359,784	2,412,586	-	642,622	304,576

^1^ https://tianchi.aliyun.com/dataset/649

^2^ https://global.jd.com

^3^ https://www.beibei.com

#### Competitors

We compare MBA with five models for multi-behavior recommendation. The competitors are divided into two categories: single-behavior and multi-behavior models. The single-behavior models are as follows.

- **MF-BPR** [7]: This approach serves as a popular optimization strategy with the assumption that positive items should receive higher scores than negative ones.
- **LightGCN** [20]: It leverages high-order connections within the user-item bipartite graph for recommendation. In particular, it removes the feature transformation and non-linear activation function from the vanilla GCN to simplify the model structure, and achieves a significant performance improvement.

The multi-behavior models are as follows.

- **MBGCN** [11]: This approach takes into account the distinct contributions of multiple behaviors to the target behavior. It learns the behavior contributions by applying Graph Convolutional Networks (GCN) on the integrated multi-behavior graph and utilizes the item-item graph to capture behavior semantics.
- **CRGCN** [9]: It employs a cascading GCN architecture to handle multi-behavior data, where features learned from one behavior are passed on to the next behavior. Additionally, this approach incorporates multi-task learning in the optimization process.
- **MB-CGCN** [10]: The model employs cascading GCN blocks to learn embeddings, where features learned by LightGCN from previous behaviors are sequentially propagated to subsequent behaviors following a feature transformation step. Ultimately, embeddings from all behaviors are aggregated to make the final prediction.

#### Evaluation metrics

We employ leave-one-out strategy [26], where one of each user’s interacted items in the target behavior (e.g., *buy*) is randomly selected for testing. We use two metrics for performance evaluations: Hit Ratio (HR@N) and normalized Discounted Cumulative Gain (nDCG@N). HR is a recall-based metric that measures whether the positive test item is recommended in the top N items in the ranking list. Normalized Discounted Cumulative Gain (nDCG) considers the position of correctly recommended items by assigning higher scores to the hits at higher positions.

#### Hyper-parameters

We set all the embedding sizes for MBA and all other methods to 64. We set the batch size to 1024. The learning rate is tuned in 1e-1, 1e-3, and 1e-4. In addition, we initialize model parameters with the Xavier initializer. For all the models using pair-wise learning loss, we randomly sample 3 negative samples for each positive sample. The number of GCN layers for each behavior is tuned in 1, 2, and 3. The behavior modeling order is *view→collect→cart→buy* for Tmall and Jdata datasets, and *view→cart→buy* for Beibei dataset, respectively. For the other hyper-parameters of the competitors, we carefully tuned them according to their original papers. Additionally, we implemented an early stopping strategy during the training phase, wherein the training process will be stopped when HR@20 metric on the validation set fails to improve over 20 epochs.

### Performance (Q1)

In this section, we report the performance comparisons between our MBA and all the competitors. The results on three datasets are shown in Table 4. The best results are highlighted in bold, and the second-best results are underlined. From the results, we see that the multi-behavior methods achieve better performance than the single-behavior models, demonstrating the benefits of leveraging auxiliary behaviors (i.e., *view* and *cart*) for the target behavior (i.e., *buy*) prediction. Overall, MBA achieves the best performance, outperforming all competitors in terms of both metrics over three datasets. The improvement across different ranges of top 10 items over the best competitor achieved up to 11.2% and 11.4% for HR@10 and nDCG@10 metrics, respectively. It demonstrates the effectiveness of our MBA model.

**Table 4:** MBA outperforms all the competitors.

Dataset	Metric	Single-behavior		Multi-behavior			
		MF-BPR	LightGCN	MBGCN	CRGCN	MB-CGCN	MBA
**Tmall**	HR@10	0.0230	0.0393	0.0549	0.0805	0.0873	**0.0934**
	nDCG@10	0.0124	0.0209	0.0285	0.0420	0.0438	**0.0488**
	HR@20	0.0316	0.0538	0.0799	0.1214	0.1295	**0.1360**
	nDCG@20	0.0144	0.0243	0.0345	0.0519	0.0541	**0.0592**
	HR@50	0.0434	0.0813	0.1285	0.1980	0.2049	**0.2127**
	nDCG@50	0.0166	0.0295	0.0438	0.0666	0.0685	**0.0739**
	HR@80	0.0541	0.0984	0.1629	0.2495	0.2522	**0.2584**
	nDCG@80	0.0183	0.0322	0.0493	0.0750	0.0762	**0.0814**
**Jdata**	HR@10	0.1850	0.2252	0.2803	0.2813	0.2904	**0.3120**
	nDCG@10	0.1238	0.1436	0.1572	0.1586	0.1614	**0.1736**
	HR@20	0.2192	0.2825	0.3603	0.3715	0.3825	**0.4038**
	nDCG@20	0.1325	0.1582	0.1790	0.1823	0.1853	**0.1981**
	HR@50	0.2652	0.3658	0.5045	0.5004	0.5173	**0.5463**
	nDCG@50	0.1417	0.1747	0.1984	0.2082	0.2124	**0.2269**
	HR@80	0.2890	0.4108	0.1807	0.5662	0.5921	**0.6203**
	nDCG@80	0.1456	0.1822	0.2098	0.2192	0.2250	**0.2394**
**Beibei**	HR@10	0.0268	0.0309	0.0373	0.1011	0.1406	**0.1442**
	nDCG@10	0.0139	0.0161	0.0193	0.0444	0.0612	**0.0627**
	HR@20	0.0427	0.0478	0.0639	0.1588	0.2105	**0.2250**
	nDCG@20	0.0179	0.0204	0.0259	0.0574	0.0775	**0.0837**
	HR@50	0.0793	0.0880	0.1287	0.2784	0.3318	**0.3446**
	nDCG@50	0.0250	0.0282	0.0386	0.0796	0.1011	**0.1051**
	HR@80	0.1075	0.1220	0.1807	0.3639	0.4093	**0.4252**
	nDCG@80	0.0297	0.0339	0.0472	0.0939	0.1143	**0.1190**

For the single-behavior models, LightGCN achieves better performance than MF-BPR. This demonstrates the benefits of GCN models in utilizing the extensive information from high-order neighbors in the user-item bipartite graph to acquire user and item embeddings for recommendation. For the multi-behavior models, MBGCN differentiates the behavior contributions to the target behavior before aggregating the embeddings learned from each behavior. CRGCN moves a step further over MBGCN by explicitly taking the cascading effects of multi-behavior into the embedding learning phase. MB-CGCN employs a feature transformation operation between GCN blocks instead of a residual design to transfer effective features from one behavior to the next. Additionally, MB-CGCN does not utilize multi-task learning in optimization, relying solely on signals from the target behavior to guide the learning process.

MBA adopts the similar cascading GCN structure as CRGCN and MB-CGCN, leveraging the benefits of explicitly utilizing cascading effects in embedding learning. However, MBA shares interaction information among GCN blocks and learns the importance of each behavior to represent embeddings. Additionally, MBA employs a behavior-aware sampling approach during model training to reflect the preference differences among behaviors. The performance improvement of MBA over MB-CGCN demonstrates the effectiveness of our design.

### Ablation study (Q2)

We conduct an ablation study to verify whether the three modules improve the performance of MBA. We analyze the contributions of each module from the following aspects.

#### Effectiveness of interaction sharing

In MBA, we transfer interaction information between two connected behaviors with embeddings learned from each behavior. To evaluate the utility of the interaction sharing module in our model, we compare our model with two variants in experiments:

- ***w/o IS:*** This variant only transfers embeddings learned from the previous behavior to the next behavior without interaction information. Each LightGCN block uses only its own adjacency matrix and does not share interaction information.
- ***with FT:*** This variant performs feature transformation to the learned embeddings before transferring to the next behavior. Each user and item embedding vector is multiplied by the transformation weight vector.

Experimental results are shown in Table 5. From the results, we observe that the performance drops after removing the interaction sharing. This demonstrates the importance of interaction sharing, which refines user preference learning and handles the issue of data sparsity. After adding feature transformation, the performance has increased. This is because feature transformation also distills useful information from an earlier behavior to help learn user and item embeddings in latter behaviors. Overall, we observe that the performance of MBA is consistently better than other methods, indicating that interaction sharing improves the prediction accuracy for the target behavior. Without interaction sharing, target behavior is not learned well due to the limited availability of information. Since *buy* interaction of Beibei dataset is not sparse compared to other datasets, its improvement is not substantial.

**Table 5:** Effects of interaction sharing in MBA. The reported performance is computed based on the top 20 results. *w/o IS* and *with FT* denote MBA without interaction sharing and with the feature transformation, respectively.

Method	Tmall		Jdata		Beibei	
	HR	nDCG	HR	nDCG	HR	nDCG
MBA *w/o IS*	0.1071	0.0425	0.3935	0.1911	0.2084	0.0759
MBA *with FT*	0.1257	0.0519	0.3555	0.1761	0.2141	0.0785
**MBA** **(proposed)**	**0.1360**	**0.0592**	**0.4038**	**0.1981**	**0.2250**	**0.0837**

#### Effectiveness of attention network

In MBA, we merge the user embeddings learned from all behaviors for the target behavior prediction by attention network. To evaluate the utility of the attention network, we compare our model with two variants in experiments:

- ***w/o att.:*** This variant removes the attention network module in MBA. It means the embeddings learned from the last GCN block are directly used for the target behavior.
- ***with agg.:*** This variant replaces the attention network with an aggregation operation. Specifically, the user and item embeddings learned from each behavior are aggregated for the target prediction.

From the results shown in Table 6, it is clear that it is important to consider different importance of behaviors.***w/o. att.*** shows the lowest performance which indicates that it is necessary to consider the embedding learned from all behaviors. It encourages the model to learn different features from each behavior.

**Table 6:** Effects of attention network in MBA. The reported performance is computed based on the top 20 results.

Method	Tmall		Jdata		Beibei	
	HR	nDCG	HR	nDCG	HR	nDCG
MBA *w/o att.*	0.0229	0.0087	0.3000	0.1511	0.1969	0.0718
MBA *with agg.*	0.1327	0.0539	0.3784	0.1863	0.2180	0.0791
**MBA** **(proposed)**	**0.1360**	**0.0592**	**0.4038**	**0.1981**	**0.2250**	**0.0837**

#### Effectiveness of behavior-based sampling

The proposed MBA uses a non-uniform sampling method to ensure that positive items are sampled from sparse but important behaviors (e.g., *buy*). To evaluate the utility of the behavior-based sampling module in our model, we compare our model with two variants in experiments:

- ***w/o BS:*** This variant removes the behavior-based sampling module in MBA. It samples positive items only from *buy* behavior, and negative items from unobserved items in *buy* behavior.
- ***with uniform:*** This variant replaces the sampling distribution with a uniform distribution. Positive items are sampled uniformly from all behaviors.

From the results shown in Table 7, it is clear that it is necessary to consider the strong user preferences in sampling. Strong user preferences during sampling are crucial because they reflect the likelihood of users engaging in specific behaviors, such as making purchases (*buy* behavior), which are typically more indicative of their preferences and intentions. By sampling positive items from behaviors with strong user preferences, the model focuses on learning from interactions that are more informative and relevant to the task of recommendation. This ensures that the model captures meaningful patterns and dependencies in the data, leading to more accurate and effective recommendations. Conversely, ignoring strong user preferences during sampling results in the model being biased towards behaviors with lower predictive power, leading to suboptimal performance and potentially inaccurate recommendations. Therefore, considering strong user preferences during sampling helps improve the overall quality and relevance of the recommendations generated by the model.

**Table 7:** Effects of behavior-based sampling in MBA. The reported performance is computed based on the top 20 results.

Method	Tmall		Jdata		Beibei	
	HR	nDCG	HR	nDCG	HR	nDCG
MBA *w/o BS*	0.0629	0.0260	0.3517	0.1675	0.1665	0.0550
MBA *with uniform*	0.0362	0.0085	0.3659	0.1777	0.1682	0.0567
**MBA** **(proposed)**	**0.1360**	**0.0592**	**0.4038**	**0.1981**	**0.2250**	**0.0837**

### Analysis (Q3)

Our model operates under the assumption that different types of user behaviors offer valuable insights into user preferences and that the sequence in which these behaviors occur (e.g., *view→cart→buy*) is significant. Specifically, later behaviors in sequences provide more specific information for refining user preferences. To validate these assumptions, we investigate the impact of multi-behavior information on recommendation performance, focusing on the number and order of behaviors.

Before presenting experimental results, we outline behavior sequences observed on platforms such as Tmall, JD, and Beibei. On Beibei, the sequence *view→cart→buy* is fixed. Conversely, on Tmall and JD, users can follow various sequences after *view*, including *collect* or *cart*, and then *buy*, or directly proceed to *buy*. Possible sequences include *view→buy*, *view→cart→buy*, *view→collect→buy*, and *view→collect→cart→buy*. Additionally, we include other behavior sequences as competitors for analysis.

**Fig 3:**
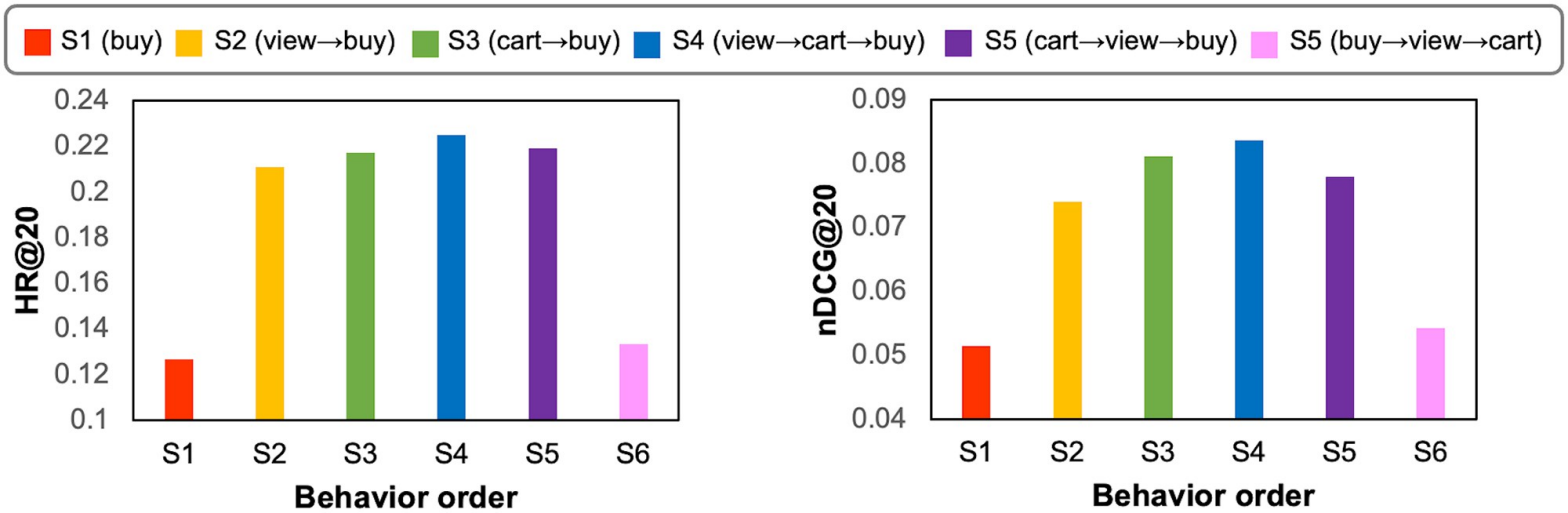
Performance comparison of MBA for different order and number of behaviors on Beibei.

The experimental results on Beibei are shown in Fig 3. Note that the behaviors must be taken in a fixed order on Beibei, e.g., *view→cart→buy*. Obviously, with more behaviors in this order, the model infers the user’s preference more accurately. Comparing S1 (*buy*), S2 (*cart*→*buy*), and S4 (*view*→*cart*→*buy*) to study the effects of behavior numbers, MBA infers user preferences more accurately with more behaviors in this order. The performance of S1 is significantly worse than that of S2 or S3, indicating the *buy* behavior is sparse, and adding the behavior helps improve the performance. Moreover, the reason why S3 performs better than S2 is that 1) *cart* behavior reveals more information about user preferences than the *view* behavior, and 2) the relationship between *buy* and *cart* is closer than the relationship between *buy* and *view*. Our model is designed to capture the relationship and significance between behaviors and leverages closer connections more effectively.

We also study the effects of behavior order on MBA by evaluating the following three behavior orders on Beibei dataset: S4 (*view*→*cart*→*buy*), S5 (*cart*→*view*→*buy*), and S6 (*buy*→*view*→*cart*). While S4 follows the correct order of the platform, the preceding two behaviors of S4 are swapped in S5, and S6 is a completely wrong order. As shown in Fig 3, S4 outperforms both S5 and S6, demonstrating the importance of correctly modeling the order of multi-behavior. The latter behavior should reveal user preferences more accurately than its previous behavior to make the embeddings of the target behavior gradually learned through the behavior blocks during model training. Our model captures user preferences step by step based on the sequence of behaviors that are often taken by users in real scenarios.

**Fig 4:**
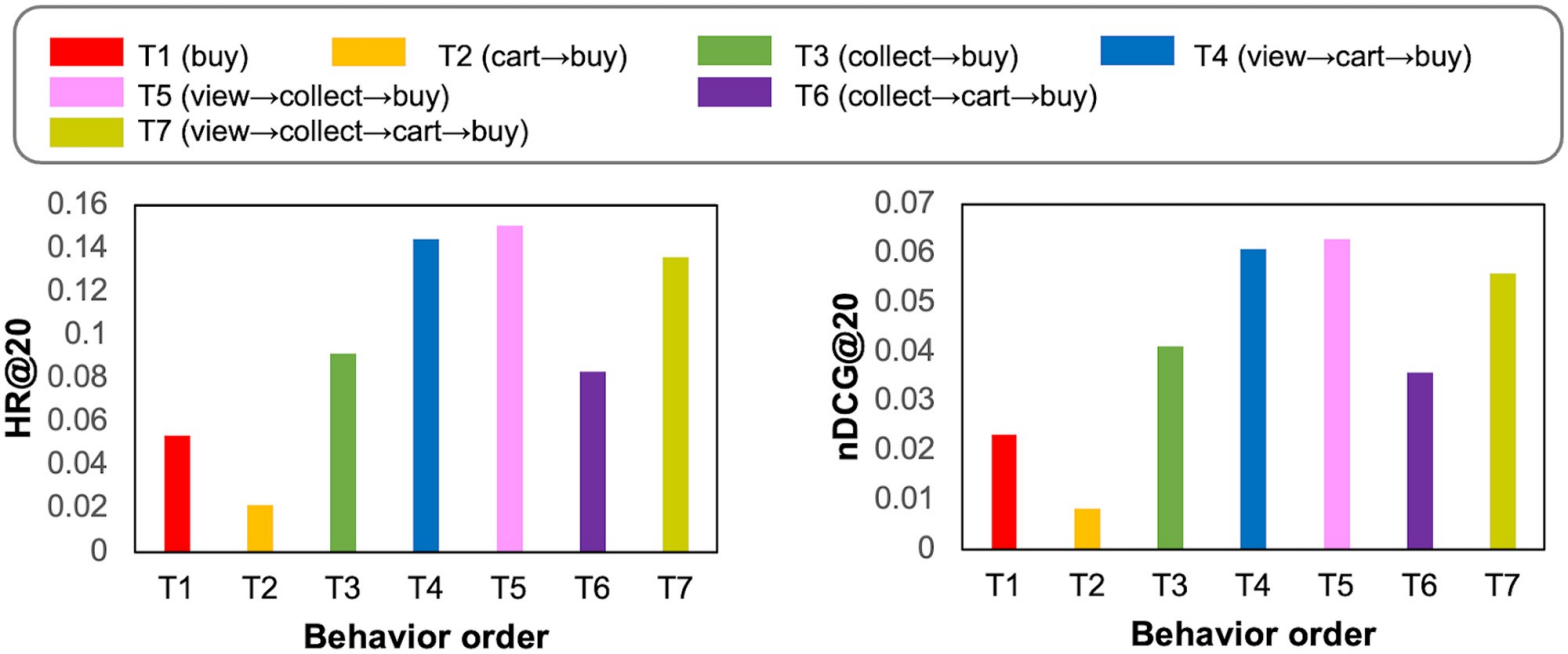
Performance comparison of MBA for different order and number of behaviors on Tmall.

**Fig 5:**
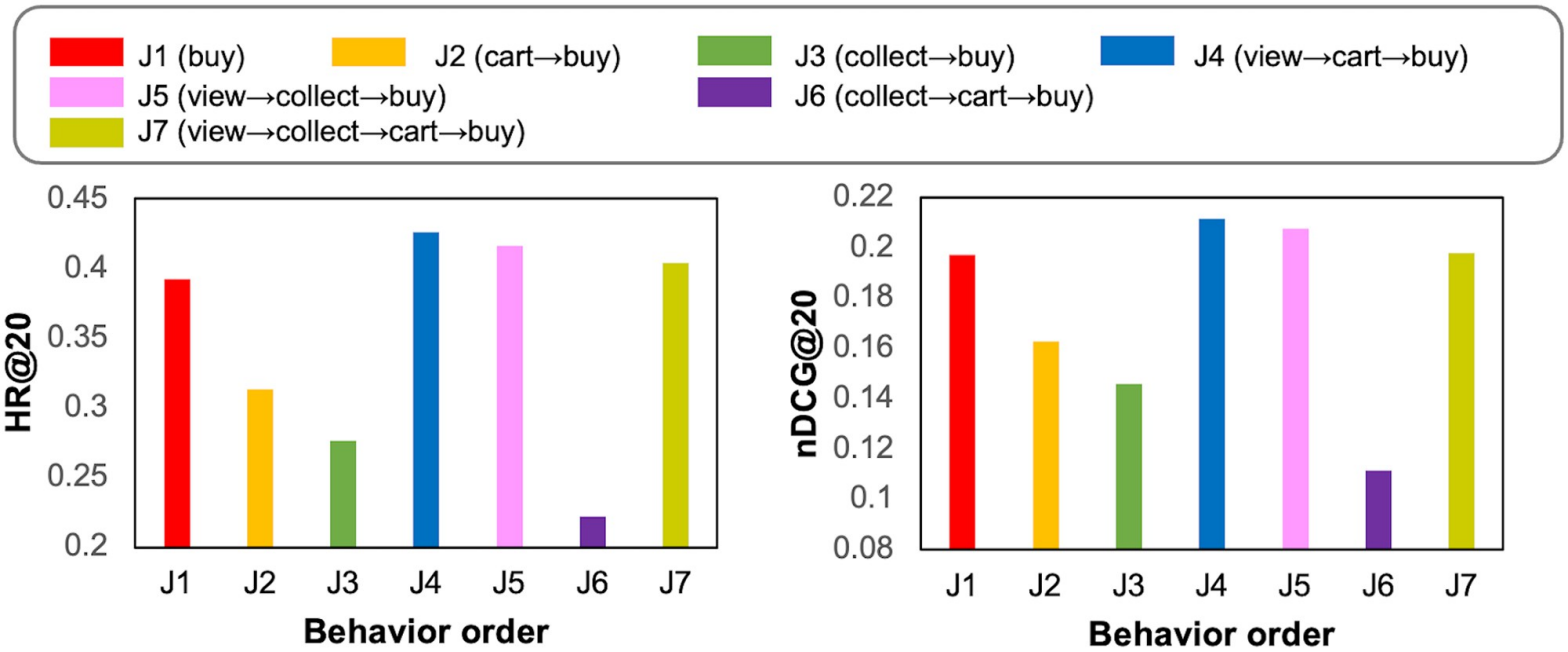
Performance comparison of MBA for different order and number of behaviors on Jdata.

The results on Tmall and Jdata datasets are shown in Figs 4 and 5. It is interesting to find that the increase in behavior numbers does not necessarily improve performance and even causes performance degradation. On Tmall, T5 (*view*→*collect*→*buy*) shows the best performance, which is better than T7 (*view*→*collect*→*cart*→*buy*). The performance of T2 (*cart*→*buy*) is worse than T1 (*buy*) because the *cart* behavior data are sparse in Tmall. As a result, users and items for such a sparse behavior hurt the embedding learning process.

By contrast, the addition of *collect* behavior improves the performance (T3 over T1, T6 over T2) for Tmall dataset. The reason is that *collect* behavior of Tmall dataset has more records (equivalent to *buy* behavior) than *cart*, so it helps mine other aspects of user preferences. The comparable performance of T4 and T5 also validates this point. Also, the performance is better when *view* behavior is the first behavior than that of other cases, demonstrating that the order of behaviors matters and *view* helps learn other behaviors in cascading effect.

For Jdata in Fig 5, the best performance is shown in J4 (*view*→*cart*→*buy*). The performance of J2 is better than J3 because *cart* contributes to *buy* more than *collect* do while the number of *collect* and *cart* behavior are similar (See Table 3). The comparable performance of J4 and J5 also validates this point. Also, J6 performs significantly low compared to other behavior orders, demonstrating that *collect* and *cart* behaviors are not much related to each other.

## Conclusions

In this paper, we introduce MBA, an accurate method for multi-behavior recommendation. Our method tackles the challenges posed by data sparsity while preserving the sequential nature of user behaviors. Also, MBA emphasise on the diverse influences of different behaviors on the target behavior prediction. sTo capture this nuanced relationship, we combine embeddings from multiple behaviors using attention weights. This allows MBA to effectively integrate information from various behaviors while giving greater importance to those that have a more significant impact on the target behavior. Moreover, we introduce a novel sampling method based on Bayesian Personalized Ranking (BPR) that optimizes the sampling process of positive and negative items in accordance with the behavioral order. By incorporating non-uniform positive item sampling, MBA enhances the sampling strategy to better reflect the sequential dependencies between behaviors.

Our experimental results demonstrate the effectiveness of MBA, with notable improvements of up to 11.2% and 11.4% in HR@10 and nDCG@10 metrics, respectively, compared to state-of-the-art competitors. In summary, MBA offers a comprehensive solution that leverages advanced techniques to provide more accurate and personalized recommendations to users.
